# IL-22 production of effector CD4^+^ T-cells is altered in SLE patients

**DOI:** 10.1186/s40001-019-0385-6

**Published:** 2019-07-22

**Authors:** Sebastian Dolff, Claudia Scharpenberg, Christof Specker, Andreas Kribben, Oliver Witzke, Benjamin Wilde

**Affiliations:** 1 Department of Infectious Diseases, University Hospital Essen, University Duisburg-Essen, 45122 Essen, Germany; 2 Department of Nephrology, University Hospital Essen, University Duisburg-Essen, 45122 Essen, Germany; 3 Department of Rheumatology and Clinical Immunology, Kliniken Essen-Mitte, 45239 Essen, Germany

**Keywords:** SLE, CD134, PD-1, IFN-γ, IL-22

## Abstract

**Background:**

Systemic lupus erythematosus (SLE) is an autoimmune disease characterized by T-cell-dependent B-cell activation and altered T-cell response. Co-stimulatory and co-inhibitory molecules regulate and exert T-cell differentiation, survival and cytokine production. CD134^+^ and PD-1^+^ T-cells in SLE patients are increased in SLE. The aim of this study was to characterize CD134^+^ and PD-1^+^CD4^+^ T-cells according to their ability to produce IFN-γ, IL-21 and IL-22 in SLE patients.

**Methods:**

Peripheral blood of 39 SLE patients and 19 healthy controls (HC) was stimulated with phorbol myristate acetate (PMA) calcium ionophore (Ca-Io). The expression of IFN-γ, IL-21 and IL-22 T-cells within the CD134^+^ and PD-1^+^ T-cells was analyzed by flow cytometry. Disease activity was assessed by SLE Disease Activity Index.

**Results:**

Peripheral unstimulated CD134^+^ and PD-1^+^ CD4^+^ T-cells were significantly increased in patients with lupus nephritis. Upon stimulation both, CD134^+^ and PD-1^+^ CD4^+^ T-cells, produced significantly less IFN-γ in SLE patients as compared to HC. The percentages of IL-22 within the CD134^+^CD4^+^ T-cells were also significantly decreased in SLE as compared to HC.

**Conclusion:**

CD134^+^ and PD-1^+^CD4^+^ T-cells have mainly a Th1 effector T-cell signature. A lower proportion produces also IL-21 and IL-22. The impaired capacity to produce IFN-γ and IL-22 in SLE patients may contribute to the pathogenesis of the disease.

## Background

Systemic lupus erythematosus (SLE) is an autoimmune disease of unknown etiology. The presence of antibodies against dsDNA is a hallmark of SLE. Although the precise pathogenesis of SLE has not been fully elucidated, T-cell-dependent B-cell hyperactivity contributes to the inflammatory pathology of SLE [1]. Crucial for antibody formation is the interaction between CD4+ T-cells and B-cells particularly in germinal centers (GCs), the site of affinity maturation followed by the subsequent generation of memory B-cells and long-lived plasma cells. Recently, the novel cytokine Interleukin (IL)-21 has been found to play a pivotal role in the differentiation and function of B-cells [2, 3].

Beside the activation of B-cells via co-stimulatory molecules, T-cells exert effector functions by the production of proinflammatory cytokines. It is commonly accepted that effector lineages can be divided into Th1, Th2 and Th17 cells according to their characteristic cytokines. Effector functions, including cytokine production, expansion and survival, are enhanced by co-stimulatory molecules such as CD134 (OX40) [4, 5]. Previously, we demonstrated an increased expression of the co-stimulatory molecule CD134 (OX40) on T-cells in systemic lupus erythematosus (SLE) patients especially with lupus nephritis [6]. Interestingly, also co-inhibitory receptors such as PD-1 were increased in CD4^+^CD25^neg^ T-cells of SLE patients [7]. Both the co-stimulatory molecule CD134 and co-inhibitory molecule PD-1 are expressed on activated T-cells and have been demonstrated to be crucial for the development of follicular T-helper-cells (T_FH_) [2]. Zander et al. demonstrated a crosstalk between PD-1 and CD134 (OX40) regarding T-helper-cell differentiation and anti-plasmodium humoral immunity in humans [8].

The CD134/OX40L axis is pivotal for the CD4^+^ differentiation toward effector T-cells. On the other hand, T-cell activation can be dampened by PD-1/PD-1L interaction [9, 10]. Interestingly, Kasagi et al. found that, in NZB/W F1 mice, a model for lupus nephritis (LN), PD-1 was predominantly expressed on kidney-infiltrating CD4^+^ T-helper cells. After blocking PD-1 with anti-PD-1 monoclonal antibodies (mAbs), there was a significant reduced mortality in the mice [11].

More recently, follicular T-helper-cells (T_FH_) were discovered characterized by IL-21 production. Several murine models indicate a crucial role of IL-21 in the pathogenesis of autoimmune diseases. Murine lupus models (BXSB-*Yaa* mice) were also investigated [12]. Interestingly, BXSB-*Yaa* mice which are IL-21R deficient show less lupus-like symptoms as compared to their wild-type BXSB-*Yaa* mice. Human studies provide further evidence that the IL-21/IL-21R pathway plays a major role in the pathogenesis of autoimmune diseases, in particular in SLE.

CD134 and PD-1 have been described as characteristic phenotypic molecules on IL-21-producing T_FH_-cells. Since we found that the effector T-cell response is altered as compared to HC, we investigated the cytokine pattern of CD134^+^ and PD-1^+^ T-cells in SLE. Therefore, the present study aims to investigate the ability of CD134^+^ and PD-1^+^CD4^+^ T-cells of human SLE patients to produce effector cytokines IFN-γ, IL-21 and IL-22 ex vivo. To investigate this, whole blood was stimulated ex vivo and the percentages of IFN-γ, IL-21 and IL-22 positive T-cells were analyzed.

## Patients and methods

### Study population

39 SLE patients with a mean age of 40 ± 14 years fulfilling at least four of the American College of Rheumatology revised criteria for SLE and 19 age and sex matched healthy controls (age 36 ± 12 years) were enrolled in this study [13]. Disease activity was assessed by SLE Disease Activity Index (SLEDAI). Thirty-two patients had inactive disease (SLEDAI score ≤ 4) and seven patients had active SLE (defined as SLEDAI score > 4). Median (range) disease activity for all patients was 2 (0–28). Thirteen patients had a biopsy-proven lupus nephritis. Follow-up visits were analyzed in six patients. Time interval between these two assessments was 9 ± 6 months. One patient did not receive any immuno-modulating medication at the time of analysis. Thirty-eight patients received immuno-modulating medication (Table 1).

**Table 1:** Baseline characteristics and medication of all patients (*n* = 39) and healthy controls (HC, *n* = 19) included

	SLE patients	HC	*p* value
Total number	39	19	
Women/men	12/1	18/1	ns
Age (years, mean ± SD)	40 ± 14	36 ± 12	ns
SLEDAI (median, range)	2 (0–28)		
Active disease (SLEDAI > 4) (*n*)	7		
Inactive disease (SLEDAI ≤ 4) (*n*)	32		
Treatment, *n*			
None	1	19	
Glucocorticoids, *n*	32		
Median dose (range), dose (mg/day)	5 (2.5–60)		
Immunosuppressive/immune-modulating			
Hydroxychloroquine, *n*	17		
Median dose (range), users (mg/day)	200 (200–400)		
Methotrexate, *n*	2		
Median dose (range), users (mg/week)	15 (15–15)		
Azathioprine, *n*	9		
Median dose (range), users (mg/day)	125 (25–250)		
MMF, *n*	14		
Median dose (range), users (mg/day)	1500 (360–2000)		
Cyclophosphamide, *n*	3		
Median dose (range), users (mg/day)	200 (100–300)		

*SLEDAI* systemic lupus erythematosus Disease Activity Index, *MMF* mycophenolate mofetil

### Sample preparation and in vitro stimulation

Methods were performed as described before [14]. In brief, sodium-heparinized venous blood was obtained from patients and healthy donors. Immediately after sampling, 200 µl blood was mixed with 200 µl RPMI1640 (Gibco Life Technologies, Paisley, UK), supplemented with 50 μg/ml gentamycin (Gibco, Scotland, UK), and aliquoted into 5-ml polypropylene tubes (BD Biosciences) (400 μl per tube). To determine the frequency of T-cell subsets, diluted whole blood was stimulated 4 h with 40 nM phorbol myristate acetate (PMA; Sigma-Aldrich, Steinheim, Germany) and 2 nM calcium ionophore (Ca-Io; Sigma-Aldrich) in the presence of 3 μM Brefeldin A. Brefeldin A was used to block intracellular transport mechanisms, thereby leading to an accumulation of cytokines in the cell. As a negative control, one sample remained without stimulation. Next, culture tubes were incubated at 37 °C, 5% CO_2_.

### Immunofluorescent staining

After stimulation, cells were washed in wash buffer [PBS, 5% Fetal Bovine Serum (FBS), 0.1% sodium azide (Merk, Germany)] and stained with Krome Orange-conjugated anti-CD3 (clone UCHT1), Pacific blue-conjugated anti-CD8 (clone B9.11), PC-7-conjugated anti-CD279 (PD-1) (clone PD-1) and PE-conjugated anti-CD134 (clone Ber-ACT35) for 15 min at room temperature. All antibodies were purchased from Beckman Coulter, Krefeld, Germany.

Cells were fixed with 100 μl *Reagent A* (Caltag Labs., An der Grab, Austria) for 10 min. After washing, the pellet was resuspended in 100 μl permeabilization *Reagent B* (Caltag Labs.) and labeled with efluor660-conjugated anti-IL21 (clone eBio3A3-N2) or with efluor660-conjugated anti-IL22 (clone 22URTI) in addition to FITC-conjugated anti-IFN-γ (clone 4S.B3), all purchased from eBioscience, for 20 min in the dark. After staining, the cells were washed and immediately analyzed on FACS-Navios flow cytometer (Beckman Coulter).

### Flow cytometric analysis

The data were analyzed using Kaluza 1.2 flow analysis software (Beckman Coulter).

As stimulation reduces surface expression of CD4 on T-cells, CD4^+^ T-cells were identified indirectly by gating on CD3-positive and CD8-negative lymphocytes [15]. The unstimulated samples were used as a guide for setting the linear gates to delineate positive and negative populations for the cytokine staining. For surface staining, isotype controls served as controls.

### Statistical analysis

Data are presented as mean ± SD unless stated otherwise. The nonparametric Mann–Whitney *U* test was used to compare data from SLE patients with that of healthy controls, and differences were considered statistically significant at two-sided *p* values less than 0.05. Paired samples were tested using the Wilcoxon signed rank test. Correlation analysis was performed using Spearman’s rank correlation coefficient.

## Results

### Increased percentages of CD134^+^CD4^+^ T-cells in SLE patients with LN

First, we studied the expression of CD134^+^CD4^+^ T-cells in unstimulated peripheral blood cells. The percentages of CD134^+^CD4^+^ T-cells were significantly increased in SLE patients as compared to healthy controls (21.4 ± 10.4% vs. 13.7 ± 5.8%; *p* = 0.002). There was no significant difference between the percentages of CD134^+^CD4^+^ T-cells in active SLE patients as compared to inactive SLE patients (24.8 ± 12.2% vs. 20.6 ± 10.1%; *p* = 0.42). The percentages of CD134^+^CD4^+^ T-cells were significantly increased in active SLE patients as compared to healthy controls (24.8 ± 12.2% vs. 13.7 ± 5.8%; *p* = 0.02) and between inactive SLE patients versus healthy controls (20.6 ± 10.1 vs. 13.7 ± 5.8%; *p* = 0.006). Next, we compared SLE patients with LN to SLE patients without LN. The percentages of CD134^+^CD4^+^ T-cells were significantly increased in SLE patients with LN as compared to SLE patients without LN and HC, respectively (25.7 ± 10.5% vs. 19.2 ± 9.9%; *p* = 0.046; 25.7 ± 10.5% vs. 13.7 ± 5.8%; *p* = 0.0007).

### Increased percentages of PD1^+^CD4^+^ T-cells in SLE patients with LN

First, we studied the expression of PD1^+^CD4^+^ T-cells in unstimulated peripheral blood cells. The percentages of PD1^+^CD4^+^ T-cells were not significantly increased in SLE patients as compared to healthy controls (35.9 ± 15.8% vs. 29.2 ± 5.8%, *p* = 0.06). There was no significant difference between active patients SLE patients as compared to inactive SLE patients (38.2 ± 23.3% vs. 35.4 ± 14.1%; *p* > 0.99) and to HC, respectively (38.2 ± 23.3% vs. 29.2 ± 5.8%; *p* = 0.73). The percentages of PD1^+^CD4^+^ T-cells were significantly increased in inactive SLE patients as compared to HC (35.4 ± 14.1% vs. 29.2 ± 5.8%; *p* = 0.04).

The percentages of PD1^+^CD4^+^ T-cells were significantly increased in SLE patients with LN as compared to HC (41.0 ± 13.6% vs. 29.2 ± 5.8%; *p* = 0.004). There was no significant difference between SLE patients with LN as compared to SLE patients without LN (41.0 ± 13.6% vs. 33.4 ± 16.4%; *p* = 0.25). There was no significant difference between SLE patients without LN and HC (33.4 ± 16.4% vs. 29.2 ± 5.8%; *p* = 0.4).

### Decreased IFN-γ and IL-22 secretion in stimulated CD134^+^CD4^+^ T-cells

**Fig. 1:**
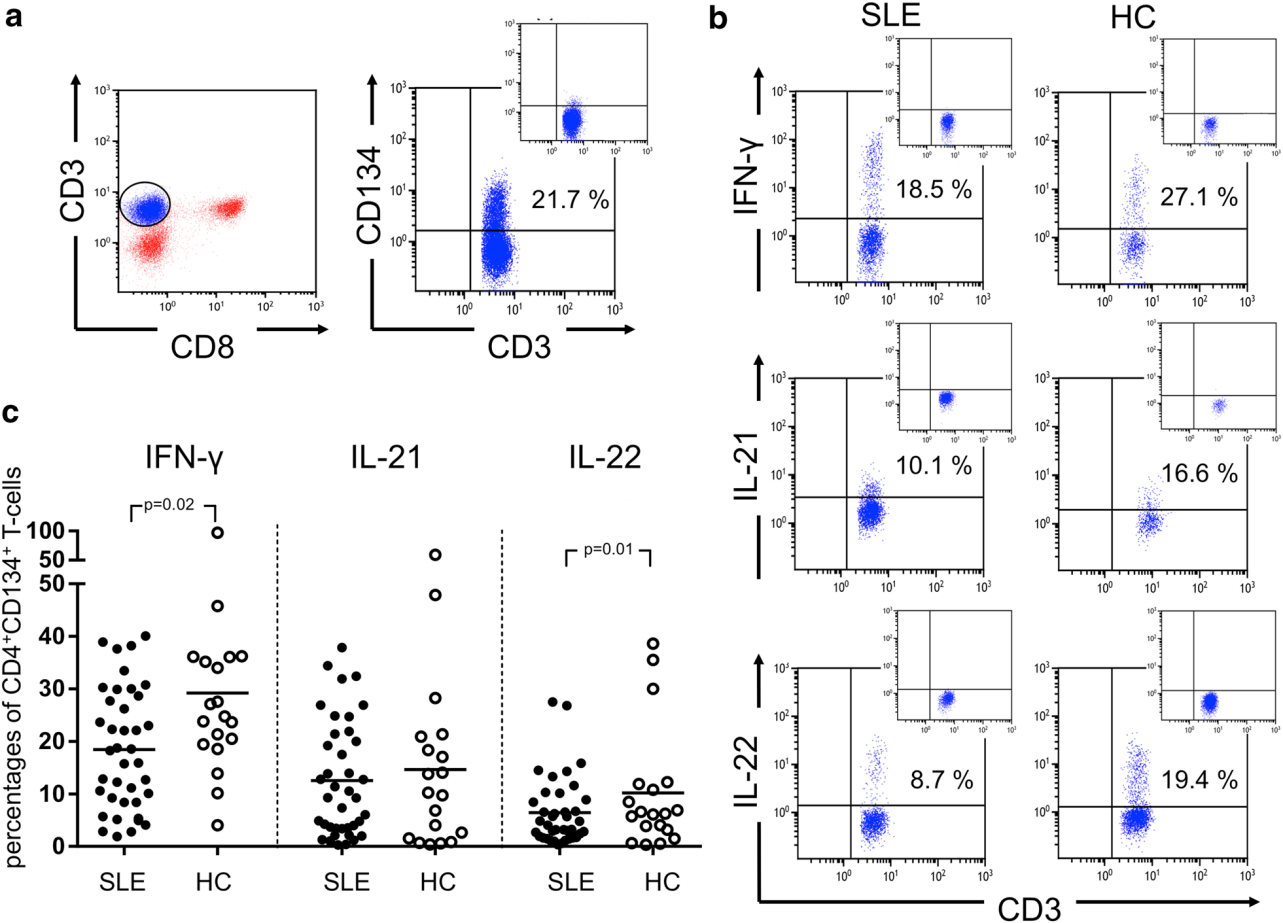
A representative dot plot of CD4^+^CD134^+^ T-cells after ex vivo stimulation with phorbol myristate acetate and calcium ionophore (**a**). CD4^+^ T-cells were defined as CD3^+^CD8^−^ T-cells. Next, CD4^+^CD134^+^ T-cells were analyzed for their ability to produce of IFN-γ, IL-21 and IL-22 in SLE patients (SLE) and healthy controls (HC). A representative dot plot of each condition is shown in **b**. The percentages of all experiments for SLE patients (*n* = 39) and HC (*n* = 19) are shown in **c**. Horizontal lines represent the mean value. Every symbol represents one measurement

Next, we studied the cytokine production of IFN-γ, IL-21 and IL-22 in CD134^+^CD4^+^ T-cells of SLE patients and HC after in vitro stimulation. The percentages of IFN-γ-producing CD134^+^CD4^+^ T-cells were significantly decreased in SLE patients as compared to HC (18.5 ± 11.5% vs. 29.2 ± 19.4%; *p* = 0.02, Fig. 1). There was no significant difference between active and inactive SLE patients (16.8 ± 10.7% vs. 18.8 ± 11.8%; *p* = 0.74) and between active SLE patients and HC (16.8 ± 10.7% vs. 29.2 ± 19.4%; *p* = 0.08). The percentages of IFN-γ-producing CD134^+^CD4^+^ T-cells in inactive patients were significantly decreased as compared to HC (18.8 ± 11.8% vs. 29.2 ± 19.4%; *p* = 0.04). There were no intraindividual differences in SLE patients between two outpatient visits (Fig. 3).

The percentages of IFN-γ-producing CD134^+^CD4^+^ T-cells were significantly increased in SLE patients with lupus nephritis as compared to patients without lupus nephritis (25.4 ± 11.4% vs. 15.0 ± 10.1%; *p* = 0.007, Fig. 4). The percentages of IFN-γ-producing CD134^+^CD4^+^ T-cells were also significantly increased in HC as compared to patients without lupus nephritis (29.2 ± 19.4% vs. 15.0 ± 10.1%; *p* = 0.001). SLE patients with lupus nephritis had no differences in the percentages of IFN-γ-producing CD134^+^CD4^+^ T-cells as compared to HC (25.4 ± 11.4% vs. 29.2 ± 19.4%; *p* = 0.99).

The percentages of IL-21-producing CD134^+^CD4^+^ T-cells were not significantly different between SLE patients and HC (11.9 ± 11.2% vs. 16.6 ± 19.0%; *p* = 0.73). There was also no difference between active and inactive SLE patients (12.5 ± 9.1% vs. 11.7 ± 11.7%; *p* = 0.63), active SLE patients and HC (12.5 ± 9.1% vs. 16.6 ± 19.0%; *p* = 0.90), and inactive SLE patients and HC (11.7 ± 11.7% vs. 16.6 ± 19.0%; *p* = 0.65). There was also no difference between the percentages of IL-21-producing CD134^+^CD4^+^ T-cells in SLE patients with lupus nephritis as compared to patients without lupus nephritis (12.5 ± 12.7% vs. 11.6 ± 10.6%; *p* = 0.89). Accordingly, no differences were found comparing patients with lupus nephritis *versus* HC, respectively (12.5 ± 12.7% vs. 16.6 ± 19.0%; *p* = 0.75).

The percentages of IL-22-producing CD134^+^CD4^+^ T-cells were significantly decreased in SLE patients as compared to HC (10.5 ± 9.0% vs. 19.0 ± 11.8%; *p* = 0.01). There was no significant difference between the percentages of IL-22-producing CD134^+^CD4^+^ T-cells in active SLE patients as compared to inactive SLE patients (8.0 ± 8.4% vs. 10.9 ± 9.2%; *p* = 0.63) and active SLE patients as compared to HC (8.0 ± 8.4% vs. 19.0 ± 11.8%; *p* = 0.06). There was a significant difference regarding the percentages of IL-22-producing CD134^+^CD4^+^ T-cells in inactive SLE patients as compared HC (10.9 ± 9.2% vs. 19.0 ± 11.8%; *p* = 0.02).

There was no significant difference comparing the percentages of IL-22-producing CD134^+^CD4^+^ T-cells in SLE patients with lupus nephritis versus patients without lupus nephritis (13.5 ± 9.7% vs. 9.0 ± 8.4%; *p* = 0.19) and HC, respectively. (13.5 ± 9.7% vs. 19.0 ± 11.8%; *p* = 0.13). There was also no significant difference with respect to the percentages of IL-22-producing CD134^+^CD4^+^ T-cells in SLE patients without lupus nephritis as compared to HC (9.0 ± 8.4% vs. 19.0 ± 11.8%; *p* = 0.008).

### Decreased IFN-γ secretion of PD1^+^CD4^+^ T-cells in SLE

**Fig. 2:**
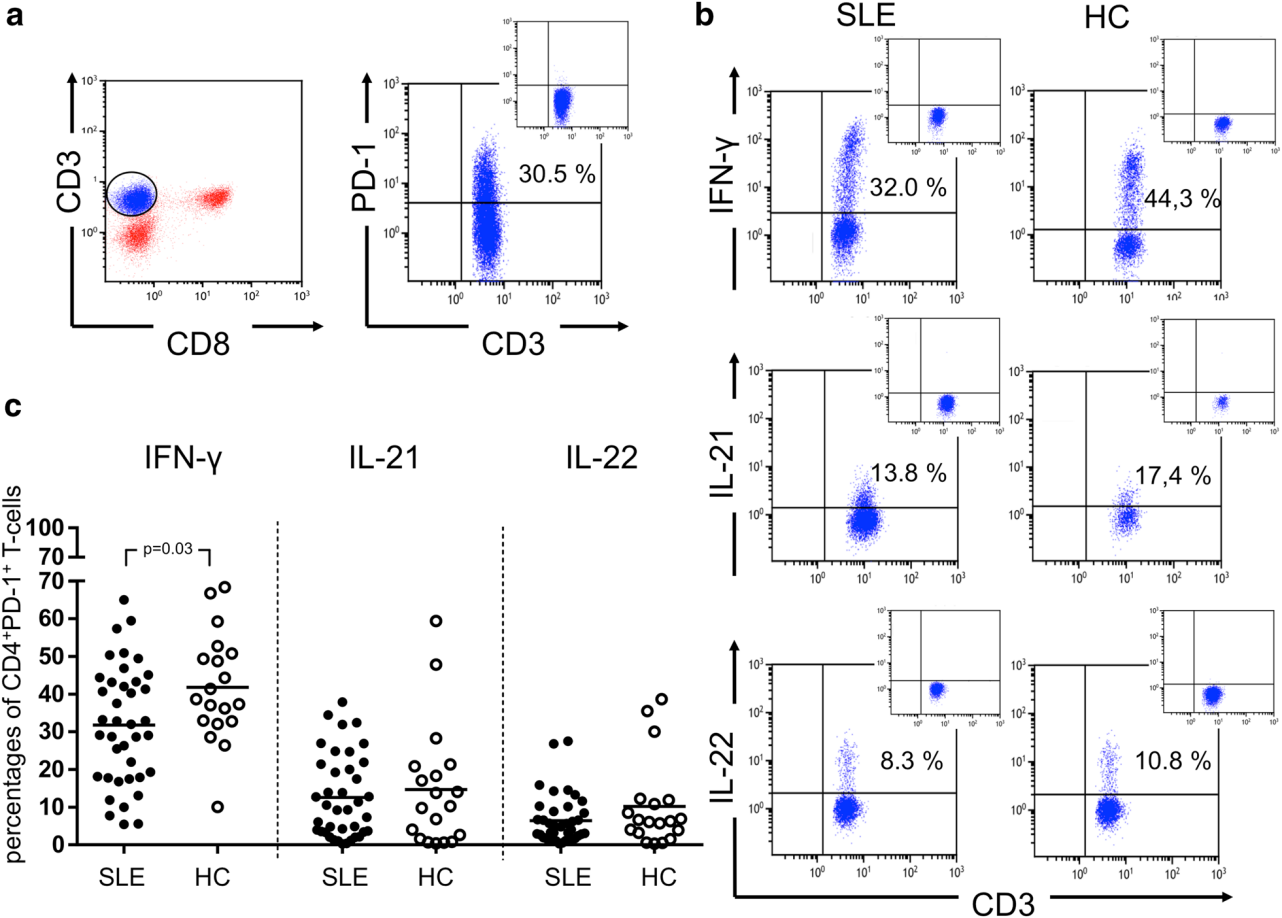
A representative dot plot of CD4^+^PD-1^+^ T-cells after ex vivo stimulation with phorbol myristate acetate and calcium ionophore (**a**). CD4^+^ T-cells were defined as CD3^+^CD8^−^ T-cells. Next, CD4^+^PD-1^+^ T-cells were analyzed for their ability to produce of IFN-γ, IL-21 and IL-22 in SLE patients (SLE) and HC (healthy controls). A representative dot plot of each condition is shown in **b**. The percentages of all experiments for SLE patients (*n* = 39) and HC (*n* = 19) are shown in **c**. Horizontal lines represent the mean value. Every symbol represents one measurement

**Fig. 3:**
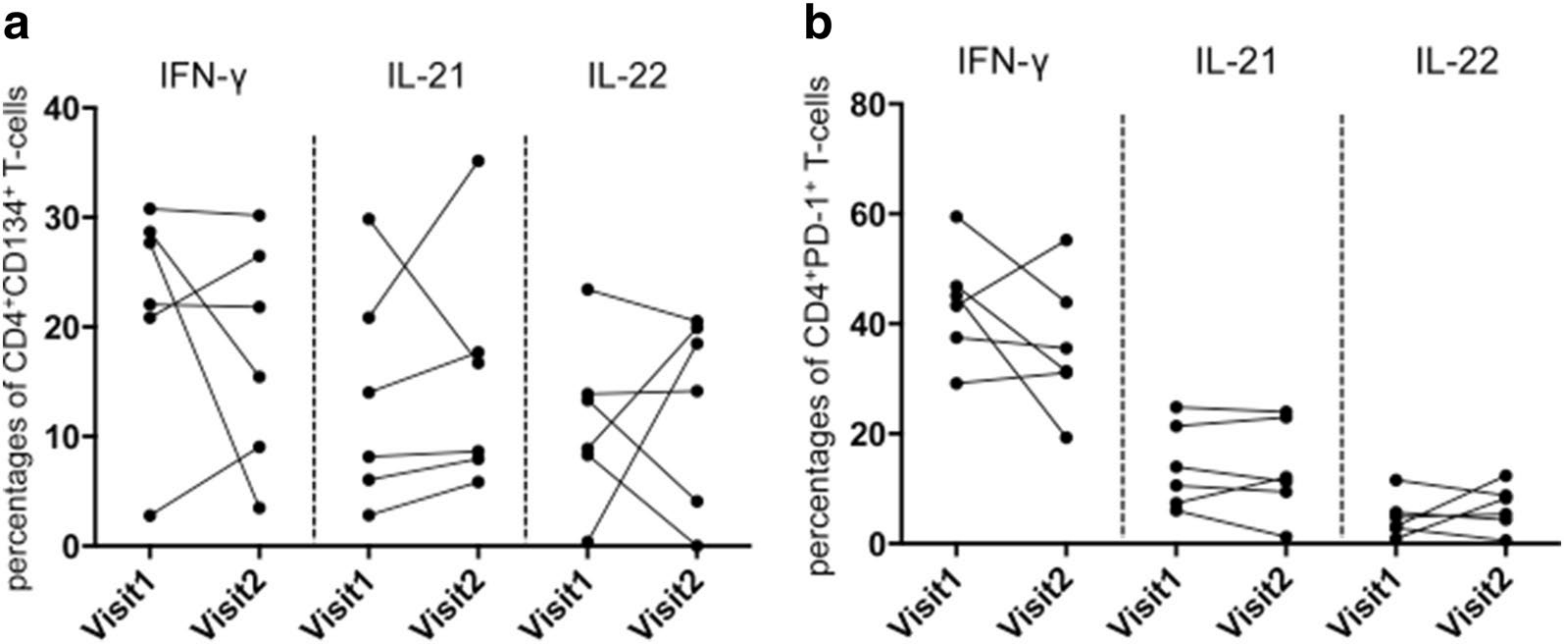
Serially analyzed SLE patients (*n* = 6) are illustrated. The time interval between these two assessments was 9 ± 6 months. *p* values were calculated using the nonparametric Wilcoxon signed rank test. No significant differences could be found

Following the analysis of PD-1 surface expression on CD4^+^ T-cells, we studied the cytokine expression of IFN-γ, IL-21 and IL-22 in PD-1^+^CD4^+^ T-cells of SLE patients and HC after in vitro stimulation. The percentages of IFN-γ-producing PD-1^+^ CD4^+^ T-cells were significantly decreased in SLE patients as compared to healthy controls (31.8 ± 15.6% vs. 41.7 ± 14.3%; *p* = 0.03, Fig. 2). The percentage of IFN-γ-producing PD-1^+^ CD4^+^ T-cells in SLE patients was stable between two outpatient visits (Fig. 3). The comparison of active versus inactive SLE patients and active SLE patients versus HC showed no significant difference regarding the percentages of IFN-γ-producing PD-1^+^CD4^+^ T-cells (38.0 ± 20.9% vs. 30.4 ± 14.3%; *p* = 0.40 and 38.0 ± 20.9% vs. 41.8 ± 14.3%; *p* = 0.75, respectively). Inactive SLE patients had significantly decreased percentages of IFN-γ-producing PD-1^+^CD4^+^ T-cells as compared to HC (30.4 ± 14.3% vs. 41.7 ± 14.3%; *p* = 0.02). There was no difference when comparing the percentages of IFN-γ-producing PD-1^+^CD4^+^ T-cells in SLE patients with LN versus SLE patients without LN (36.1 ± 14.0% vs. 29.6 ± 16.2%; *p* = 0.20); likewise, no difference was found between SLE patients with LN and HC (36.1 ± 14.0% vs. 41.8 ± 14.3%; *p* = 0.40). SLE patients without LN had significantly decreased percentages of IFN-γ-producing PD-1^+^CD4^+^ T-cells as compared to HC (29.6 ± 16.2% vs. 41.8 ± 14.3%; *p* = 0.02).

**Fig. 4:**
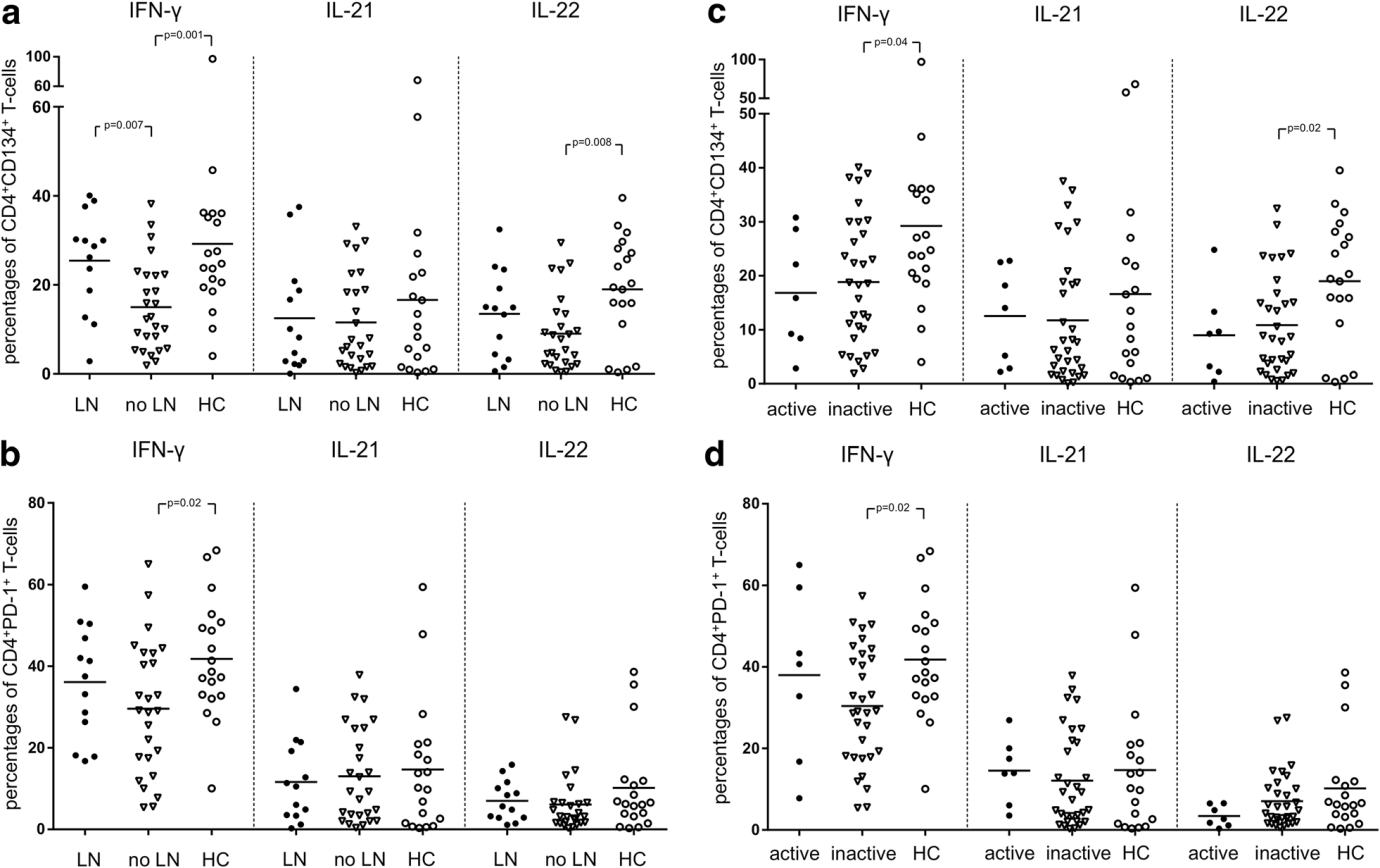
The percentages of IFN-γ, IL-21 and IL-22 in ex vivo stimulated CD134^+^CD4^+^ T-cells (**a**) and PD-1^+^CD4^+^ T-cells (**b**) in SLE patients with lupus nephritis (LN) (*n* = 13), SLE patients without lupus nephritis (no LN) (*n* = 26) and healthy controls (HC) (*n* = 19) are shown. SLE patients with active and inactive disease as compared to HC are shown in **c** for CD134^+^CD4^+^ T-cells and PD-1^+^CD4^+^ T-cells (**d**). Horizontal lines represent the mean value. Every symbol represents a measurement

The percentages of IL-21-producing PD-1^+^ CD4^+^ T-cells were not significantly different in SLE patients as compared to healthy controls (12.6 ± 11.0% vs. 14.7 ± 16.2%; *p* = 0.91, Fig. 2). The percentages of IL-21-producing PD-1^+^CD4^+^ T-cells were also not significantly different in active SLE patients as compared to inactive SLE patients (14.5 ± 8.0% vs. 12.11 ± 11.6%; *p* = 0.30) and as compared to HC (14.5 ± 8.0% vs. 14.7 ± 16.2%; *p* = 0.53). There was also no difference in the production of IL-21 in PD-1^+^ CD4^+^ T-cells of SLE patients with LN as compared to SLE patients without LN (11.6 ± 10.1% vs. 13.0 ± 11.6%; *p* = 0.79). Similarly, there was no statistically significant difference comparing SLE patients with LN versus HC (11.6 ± 10.1% vs. 14.7 ± 16.2%; *p* = 0.98, Fig. 4).

The fraction of IL-22-producing PD-1^+^ CD4^+^ T-cells was not significantly different in SLE patients as compared to HC (6.4 ± 6.5% vs. 10.2 ± 11.6%; *p* = 0.26). The percentages of IL-22-producing PD-1^+^ CD4^+^ T-cells were also not significantly different comparing active and inactive patients (3.4 ± 2.6% vs. 7.1 ± 6.8%; *p* = 0.17) as well as active patients versus HC, respectively (3.4 ± 2.6% vs. 10.2 ± 11.6%; *p* = 0.10). There were no differences between all other groups analyzed.

### Influence of immunosuppressive treatment

**Fig. 5:**
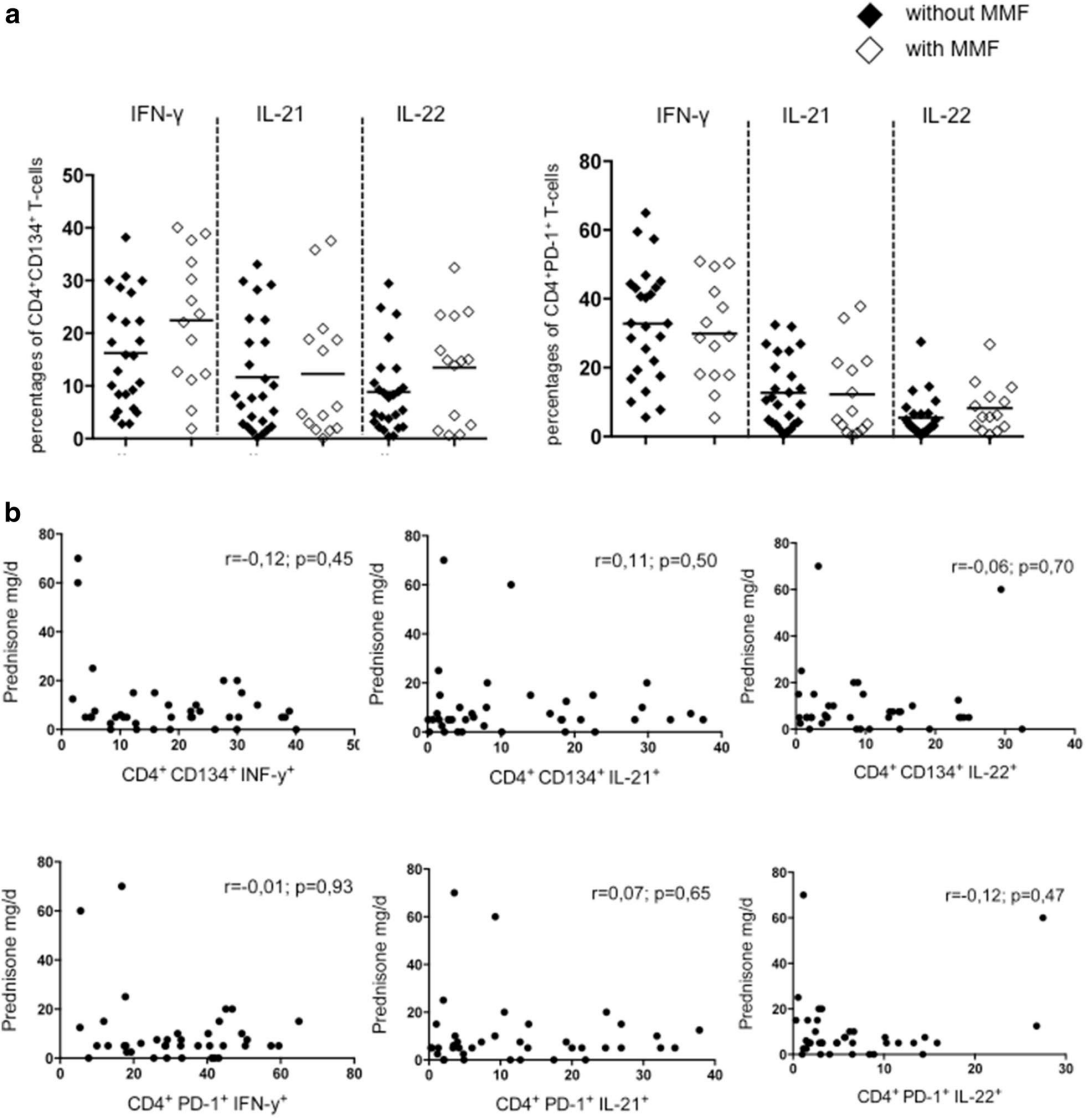
The percentages of IFN-γ, IL-21 and IL-22 in ex vivo stimulated CD134^+^CD4^+^ T-cells and PD-1^+^CD4^+^ T-cells in SLE patients receiving mycophenolate mofetil (*n* = 14) versus SLE patients without mycophenolate mofetil (*n* = 25) (**a**). To assess the influence of glucocorticoids, a correlation analysis was performed in all SLE patients (*n* = 39) (**b**)

To analyze the cytokine-secreting capacity of CD134^+^ and PD-1^+^CD4^+^ T-cell subsets dependent on the immunosuppressive treatment, we stratified the patients in two groups. We compared patients who received MMF to patients with an immunosuppressive regimen without MMF. There was no significant difference between these groups regarding effector cytokine production (IFN-γ, IL-21 and IL-22) within CD134^+^ and PD-1^+^CD4^+^ T-cells (Fig. 5a). To investigate the role of prednisone, we correlated the daily dose of prednisone in mg/day with these T-cell subsets. There was no significant correlation found in these groups (Fig. 5b).

## Discussion

The present study confirms our previous observation that CD134^+^CD4^+^ T-cells are increased in the peripheral blood of patients with systemic lupus erythematosus as compared to HC [6]. The proportion is even more increased in patients with lupus nephritis and has been found in inflamed tissue of lupus nephritis biopsies [16]. The observation by Aten et al. that CD134L is abundantly present in proliferative lupus nephritis predominantly along the epithelial side of the glomerular capillary wall highlights the CD134-CD134L pathway as a potential therapeutic target [17]. In this study, we could also find increased expression of PD-1 on peripheral CD4^+^ T-cells in lupus nephritis patients. A tendency of increased expression of PD-1 on peripheral CD4^+^ T-cells was found in our previous study which was significant different most likely due to the lower numbers of LN patients included (*n* = 7) in contrast to 13 patients in the present study [7].

The significance of CD134 for effector functions has been shown by the observation that treatment with a stimulatory anti-CD134 antibody enhances T-cell expansion and differentiation to effector cells in mice [18, 19]. This stimulation, apparently, promotes the secretion of IFN-γ and the upregulation of various interleukin (IL)-receptors which might lead subsequently to cytokine-mediated kidney cell damage [20]. Interestingly, upon stimulation CD134^+^ and PD-1^+^CD4^+^ T-cells produce significant lower amounts of IFN-γ in SLE patients as compared to HC. This decrease was not associated with lupus nephritis or active disease. Notwithstanding, immunosuppressive treatment could have impact on the capacity to produce effector cytokines. In our present study there was no correlation between the percentages and the daily dose of glucocorticoids. Patients with more intense immunosuppressive treatment did also not show significantly lower capacities to produce IFN-γ. However, individualized immunosuppressive treatment regimens may hamper the interpretation. Another limitation of the present study is the analysis of stimulated CD3^+^CD8^−^ T-cells. This population consists mainly of CD4^+^ T-cells but significant proportions of γδ T-cells have been reported in particular after treatment [21]. The percentages of IFN-γ and IL-17 within this subset are relatively low. Thus, the exclusion of this subset would probably not change the current results significantly.

Both CD134^**+**^CD4^+^ T-cell and PD-1^**+**^ CD4^+^ T-cell subsets were characterized by a Th1 cytokine pattern. IL-21 production was surprisingly not different between SLE patients and HC and relatively low with about 15%. This may indicate that CD134 and PD-1 T-cells express IL-21^+^ in the germinal center (GC) but in peripheral blood the main cytokine response is IFN-γ.

In contrast to our results, Jacquemin et al. reported that IL-12 signals cooperate with OX40 signals to increase the expression of CXCR5 and IL-21 by memory Th-cells [22].

Th22 cell are described as a novel T-cell lineage producing the signature cytokine IL-22. IL-22has been shown to be increased in the peripheral blood of SLE patients with sole lupus skin disease and to be decreased in lupus nephritis patients [23]. The present study reveals also a decrease of IL-22 within the CD134^+^CD4^+^ T-cells. Moreover, the mRNA levels of IL-22 were decreased in the urine of LN patients with proliferative glomerulonephritis and inversely correlated with the histological activity index [24]. The ligation of CD134 and CD134L along the epithelial side of the glomerular capillary might enhance Th22 cell migration to the inflamed kidney.

## Conclusion

The present study demonstrates the altered cytokine pattern of CD134^+^ and PD-1^+^CD4^+^ effector T-cells in patients with systemic lupus erythematosus. The capacity to produce IFN-γ, IL-21 and IL-22 is impaired. Both effector subsets are mainly characterized by Th-1 response while T_FH_- and Th-22 cytokines are also produced at lower levels. These results confirm that co-stimulatory and co-inhibitory molecules remain an attractive target for immune-modulatory therapies.

## Data Availability

The datasets analyzed during the current study is available from the corresponding author on reasonable request.

## Abbreviations

Ca-Io: calcium ionophore
GC: germinal center
HC: healthy controls
IL: interleukin
LN: lupus nephritis
MMF: mycophenolate mofetil
PMA: phorbol myristate acetate
SLE: systemic lupus erythematosus
SLEDAI: SLE Disease Activity Index
T_FH_: follicular T-helper-cells

## References

[1] Dorner T, Giesecke C, Lipsky PE (2011). Mechanisms of B cell autoimmunity in SLE. Arthritis Res Ther.

[2] Crotty S (2011). Follicular helper CD4 T cells (TFH). Annu Rev Immunol.

[3] Blanco P, Ueno H, Schmitt N (2016). T follicular helper (Tfh) cells in lupus: activation and involvement in SLE pathogenesis. Eur J Immunol.

[4] Croft M (2010). Control of immunity by the TNFR-related molecule OX40 (CD134). Annu Rev Immunol.

[5] Walker LS, Gulbranson-Judge A, Flynn S, Brocker T, Raykundalia C, Goodall M (1999). Compromised OX40 function in CD28-deficient mice is linked with failure to develop CXC chemokine receptor 5-positive CD4 cells and germinal centers. J Exp Med.

[6] Patschan S, Dolff S, Kribben A, Durig J, Patschan D, Wilde B (2006). CD134 expression on CD4+ T cells is associated with nephritis and disease activity in patients with systemic lupus erythematosus. Clin Exp Immunol.

[7] Dolff S, Quandt D, Feldkamp T, Jun C, Mitchell A, Hua F (2014). Increased percentages of PD-1 on CD4+ T cells is associated with higher INF-gamma production and altered IL-17 production in patients with systemic lupus erythematosus. Scand J Rheumatol.

[8] Zander RA, Obeng-Adjei N, Guthmiller JJ, Kulu DI, Li J, Ongoiba A (2015). PD-1 co-inhibitory and OX40 co-stimulatory crosstalk regulates helper T cell differentiation and anti-plasmodium humoral immunity. Cell Host Microbe.

[9] Okazaki T, Maeda A, Nishimura H, Kurosaki T, Honjo T (2001). PD-1 immunoreceptor inhibits B cell receptor-mediated signaling by recruiting src homology 2-domain-containing tyrosine phosphatase 2 to phosphotyrosine. Proc Natl Acad Sci USA.

[10] Latchman Y, Wood CR, Chernova T, Chaudhary D, Borde M, Chernova I (2001). PD-L2 is a second ligand for PD-1 and inhibits T cell activation. Nat Immunol.

[11] Kasagi S, Kawano S, Okazaki T, Honjo T, Morinobu A, Hatachi S (2010). Anti-programmed cell death 1 antibody reduces CD4+PD-1+ T cells and relieves the lupus-like nephritis of NZB/W F1 mice. J Immunol.

[12] Bubier JA, Sproule TJ, Foreman O, Spolski R, Shaffer DJ, Morse HC (2009). A critical role for IL-21 receptor signaling in the pathogenesis of systemic lupus erythematosus in BXSB-Yaa mice. Proc Natl Acad Sci USA.

[13] Hochberg MC (1997). Updating the American College of Rheumatology revised criteria for the classification of systemic lupus erythematosus. Arthritis Rheum.

[14] Dolff S, Abdulahad WH, Westra J, Doornbos-van der Meer B, Limburg PC, Kallenberg CG (2011). Increase in IL-21 producing T-cells in patients with systemic lupus erythematosus. Arthritis Res Ther.

[15] Pelchen-Matthews A, Parsons IJ, Marsh M (1993). Phorbol ester-induced downregulation of CD4 is a multistep process involving dissociation from p56lck, increased association with clathrin-coated pits, and altered endosomal sorting. J Exp Med.

[16] Dolff S, Quandt D, Wilde B, Feldkamp T, Hua F, Cai X (2010). Increased expression of costimulatory markers CD134 and CD80 on interleukin-17 producing T cells in patients with systemic lupus erythematosus. Arthritis Res Ther.

[17] Aten J, Roos A, Claessen N, Schilder-Tol EJ, Ten Berge IJ, Weening JJ (2000). Strong and selective glomerular localization of CD134 ligand and TNF receptor-1 in proliferative lupus nephritis. J Am Soc Nephrol JASN.

[18] Lathrop SK, Huddleston CA, Dullforce PA, Montfort MJ, Weinberg AD, Parker DC (2004). A signal through OX40 (CD134) allows anergic, autoreactive T cells to acquire effector cell functions. J Immunol.

[19] Huddleston CA, Weinberg AD, Parker DC (2006). OX40 (CD134) engagement drives differentiation of CD4+ T cells to effector cells. Eur J Immunol.

[20] Williams CA, Murray SE, Weinberg AD, Parker DC (2007). OX40-mediated differentiation to effector function requires IL-2 receptor signaling but not CD28, CD40, IL-12Rbeta2, or T-bet. J Immunol.

[21] Ma H, Yuan Y, Zhao L, Ye Z, Xu J, Li M (2016). Association of gammadelta T cell compartment size to disease activity and response to therapy in SLE. PLoS ONE.

[22] Jacquemin C, Schmitt N, Contin-Bordes C, Liu Y, Narayanan P, Seneschal J (2015). OX40 ligand contributes to human lupus pathogenesis by promoting T follicular helper response. Immunity.

[23] Yang XY, Wang HY, Zhao XY, Wang LJ, Lv QH, Wang QQ (2013). Th22, but not Th17 might be a good index to predict the tissue involvement of systemic lupus erythematosus. J Clin Immunol.

[24] Yang X, Gao Y, Wang H, Zhao X, Gong X, Wang Q (2014). Increased urinary interleukin 22 binding protein levels correlate with lupus nephritis activity. J Rheumatol.

